# Can machine learning models predict maternal and newborn healthcare providers’ perception of safety during the COVID-19 pandemic? A cross-sectional study of a global online survey

**DOI:** 10.1186/s12960-022-00758-5

**Published:** 2022-08-19

**Authors:** Bassel Hammoud, Aline Semaan, Imad Elhajj, Lenka Benova

**Affiliations:** 1 Biomedical Engineering Program, Faculty of Medicine-Maroun Semaan Faculty of Engineering and Architecture, American University of Beirut, Beirut, Lebanon; 2 Department of Public Health, Institute of Tropical Medicine, Antwerp, Belgium; 3 Electrical and Computer Engineering Department, Maroun Semaan Faculty of Engineering and Architecture, American University of Beirut, Beirut, Lebanon

**Keywords:** Healthcare providers, COVID-19, Machine learning, Maternal health

## Abstract

**Background:**

Maternal and newborn healthcare providers are essential professional groups vulnerable to physical and psychological risks associated with the COVID-19 pandemic. This study uses machine learning algorithms to create a predictive tool for maternal and newborn healthcare providers’ perception of being safe in the workplace globally during the pandemic.

**Methods:**

We used data collected between 24 March and 5 July 2020 through a global online survey of maternal and newborn healthcare providers. The questionnaire was available in 12 languages. To predict healthcare providers’ perception of safety in the workplace, we used features collected in the questionnaire, in addition to publicly available national economic and COVID-19-related factors. We built, trained and tested five machine learning models: Support Vector Machine (SVM), Random Forest (RF), XGBoost, CatBoost and Artificial Neural Network (ANN) for classification and regression. We extracted from RF models the relative contribution of features in output prediction.

**Results:**

Models included data from 941 maternal and newborn healthcare providers from 89 countries. ML models performed well in classification and regression tasks, whereby RF had 82% cross-validated accuracy for classification, and CatBoost with 0.46 cross-validated root mean square error for regression. In both classification and regression, the most important features contributing to output prediction were classified as three themes: (1) information accessibility, clarity and quality; (2) availability of support and means of protection; and (3) COVID-19 epidemiology.

**Conclusion:**

This study identified salient features contributing to maternal and newborn healthcare providers perception of safety in the workplace. The developed tool can be used by health systems globally to allow real-time learning from data collected during a health system shock. By responding in real-time to the needs of healthcare providers, health systems could prevent potential negative consequences on the quality of care offered to women and newborns.

**Supplementary Information:**

The online version contains supplementary material available at 10.1186/s12960-022-00758-5.

## Introduction

In the last 20 years, coronaviruses have caused several outbreaks, such as severe acute respiratory syndrome (SARS) in 2002, and middle east respiratory syndrome (MERS) in 2012 [1]. During December 2019, several cases of respiratory distress were reported in Wuhan City in China, due to a novel coronavirus (SARS-CoV-2). Following an exponential increase in the number of cases, the World Health Organization (WHO) declared COVID-19 a “global health emergency” on January 2020, and in March 2020 a “pandemic” [2]. Globally, as of 18 May 2022, there have been more than 520 million confirmed cases of COVID-19, including more than 6.2 million deaths [3].

The impact of the COVID-19 pandemic is not limited to physical health; it has repercussions on the psychological, social, and economic level, including on healthcare infrastructure [4]. Healthcare providers are particularly vulnerable to the risks associated with COVID-19, with several studies reporting an increased prevalence of depression, anxiety, insomnia and psychological distress [5]. According to a study among healthcare workers in China, this is associated with many factors, including the rapidly increasing number of cases and deaths, the quick spread of the virus, the overwhelming workload, lack of access to personal protective equipment (PPE), absence of clear guidelines especially at the beginning of the pandemic, and fear of spreading the virus to family members [6]. A longitudinal study among healthcare providers in Argentina shows a worsening of mental health among providers who expressed concern about infection with COVID-19 [7].

Maternal and newborn healthcare providers faced personal and professional challenges during the COVID-19 pandemic as they continued to provide essential health services to women, babies and families. In the United Kingdom (UK) and Italy, many deaths were reported among midwives due to COVID-19 [8]. Few studies were conducted to document those challenges and experiences, with the majority being from high- and middle-income countries [9–16]; including one global survey of providers caring specifically for small and sick newborns [17]. With the inadequate supply of PPE, which in many settings was prioritised for healthcare providers working in COVID-19 treatment wards, maternal and newborn healthcare providers worried about their own health and were concerned over occupational exposure to COVID-19 in the workplace, and transmitting the infection to patients, family and friends [9, 12, 14, 17]. A survey conducted by the Royal College of Midwives in the UK revealed that more than half of the midwives did not feel safe to conduct home visits in April 2020 [18]. Loss of social support and increased levels of stress and anxiety were common among maternal and newborn healthcare providers during this period [13, 15, 17]. In Nigeria, the majority of maternal and newborn healthcare providers worried about stigmatisation or discrimination as a result of their potential exposure to COVID-19, and 87% experienced work-related burnout [9]. Maternal and newborn healthcare providers were additionally overwhelmed by the amount of new information and guidelines that were frequently changing in the early phase of the pandemic [17]. This was not universal, however, and in some settings, healthcare providers reported being adequately informed [15, 16].

Based on the above summary of the literature, we hypothesise that factors at various levels could influence maternal and newborn healthcare providers’ wellbeing and their perception of safety during the COVID-19 pandemic. A first factor is the perceived ability to protect themselves against infection (e.g. through the availability of PPE) [6, 9, 12, 14, 17]. Second, healthcare providers’ perceived risk of infection with COVID-19 can influence their wellbeing, and this risk can be reflected by the number of confirmed COVID-19 cases and deaths in the country and the numbers of cases and deaths among healthcare providers themselves [6, 8, 18]. Third, the wellbeing of healthcare providers depends on the perceived adequacy of information guidelines [6, 17].

During the COVID-19 pandemic, hundreds of machine learning (ML) models were built and applied to address various issues related to the pandemic, including automated diagnosis by extracting COVID-19 specific patterns from chest X-rays and CT-scans, predicting epidemiologic outbreaks, discovering therapeutics and designing novel vaccines [19–21], and to predict the effect of non-pharmaceutical interventions on the COVID-19 epidemiology globally [22]. Some studies applied these techniques to tackle mental health issues and psychological stressors for the general population, including for healthcare providers during the pandemic [23, 24]. However, the majority of studies assessing the mental health of healthcare providers (using ML or not) included individuals from single countries (e.g. China [25], USA [26], Turkey [27]) or from high-income countries [28]. In India, a study is planned to predict burnout among healthcare providers due to the COVID-19 pandemic using ML [29]. No study has used data from a diversity of country income groups.

The objective of this study is to use ML algorithms to create a predictive tool based on main drivers contributing to maternal and newborn healthcare providers’ perception of being safe in the workplace globally and compare its performance to standard statistical models. Specifically, we aim to identify the most salient factors contributing to perception of safety among maternal and newborn healthcare providers.

## Methods

### Study design and data collection

This cross-sectional study uses data collected between 24 March 2020 and 5 July 2020, during the first round of a global online survey of maternal and newborn healthcare providers during the COVID-19 pandemic. The survey targeted various cadres of maternal and newborn healthcare providers, including midwives, nurse-midwives, nurses, obstetricians/gynaecologists, neonatologists and paediatricians, among others. Participants were invited to complete the survey through personal and professional networks, and social media channels (e.g. Twitter, Facebook, WhatsApp groups, etc.). Additional details about the study design and sampling are available elsewhere [30]. The questionnaire was available in 12 languages (Arabic, Chinese, Dutch, English, French, German, Italian, Japanese, Kiswahili, Portuguese, Russian and Spanish), and it was published online using KoboToolbox’s online data collection feature [31].

### Questionnaire and definitions

The questionnaire was developed by an international multidisciplinary team including health professionals, experts in health systems, maternal and newborn health epidemiologists and public health researchers. The questionnaire consisted of four main modules including questions about (1) respondents’ background information and characteristics of the facilities where they worked; (2) preparedness for the COVID-19 pandemic, including access to information and training; (3) facility-level response to the COVID-19 pandemic including setting-up screening areas and PPE availability; and (4) healthcare providers’ work-related experiences since the start of the COVID-19 pandemic, including stress levels and concerns. The full questionnaire is available on the study website [32].

In the disciplines of computer engineering/science and public health, different terminologies are used to describe similar concepts. Throughout this manuscript, we use terminologies adopted in computer engineering/science. The term “output” is used in computer engineering/science disciplines and is equivalent to the term “dependent variable” used in public health. It refers to the predicted factor which is a respondent’s perception of feeling protected from infection with COVID-19 in the workplace at the time of the survey. This was collected on a 5-point Likert scale: (1) not at all protected, (2) minimally protected, (3) some protection, (4) well protected, and (5) completely protected. The term “features” or “model inputs” used in computer engineering/science is equivalent to the term “explanatory variables” or “independent variables” in public health and refers to the factors that are fixed and used to predict/explain the output (listed in Additional file 1).

A few features were added to the dataset after data collection was completed. These capture characteristics of the countries where respondents worked. The country income level variable (high-income, middle-income, low-income countries) was defined using the World Bank classification of the worlds’ economies (according to 2019 gross national income) [33]. Another economic indicator from the World Bank database was the gross domestic product per capita expressed in current international dollars for the year 2019 [34]. The national estimates of the maternal mortality ratio (MMR) were added based on the WHO’s 2017 estimates [35]. National level events relevant to the COVID-19 pandemic, including cumulative number of cases and deaths, lockdowns, curfews, domestic and foreign travel bans, mask mandates were sourced from the Oxford COVID-19 Government Response Tracker, Blavatnik School of Government, University of Oxford [36]. These data were merged with the survey data based on the country and the date of data collection. The complete list of features, including their data sources (*n* = 71 features; 41 from survey, 23 from the Government Response Tracker, 4 from the WHO COVID-19 dashboard, 2 from the World Bank database; 1 from the WHO estimates on maternal mortality) is provided in Additional file 1.

### Data management

#### Missing answers

Features collected through the survey to which more than 30% of the respondents did not provide answers were removed from the dataset. Furthermore, respondents who had at least one missing feature were excluded from analysis. The remaining dataset contained a total of 941 respondents out of 1641 submissions originally made.

#### Data pre-processing

To deal with class imbalance, the data were augmented using Synthetic Minority Oversample Technique (SMOTE) from the Imbalanced-Learn Library [37]. The technique consists of oversampling examples in the minority class (the class of the output with fewest individuals), by randomly selecting an instance from this class, choosing a certain number of nearest neighbours to that instance and interpolating new datapoints between the selected neighbours in the feature space. This leads to an augmented dataset with balanced classes of the output. The features were then appropriately encoded based on their type: one-hot encoding [38] for categorical features and ordinal encoding for ordinal features. Numerical features were standardised using Scikit-learn Standard Scaler (features become centred around their mean with a unit standard deviation), to allow faster convergence of the models. The dataset was then randomly split into a training set (80% of sample) used to train models and a testing set (20%) used to test the performance of different models for new respondents.

### Data analysis

#### Machine learning models

Two different approaches were used to predict the output: classification and regression. In classification models, the output was employed as a categorical variable, and the goal was to train the model to predict a discrete class of the output to which the respondent belongs. In regression models, the output feature was employed as a continuous variable, and the goal was to predict a decimal score from 1 to 5 reflecting the output.

We built, trained and tested five machine learning models, and compared them to the conventional statistical methods (Linear Regression and Logistic Regression). The 5 ML algorithms (sequence of steps that lead to the model when implemented on the data) used are: Support Vector Machine (SVM), Random Forest (RF), XGBoost, CatBoost and Artificial Neural Network (ANN). These models were chosen due to their predictive abilities in healthcare settings in general and in public health and mental health applications in particular [39–43]. The models are described individually in detail in Additional file 2.

#### Hyperparameters tuning

To build a robust ML model, the optimal set of “hyperparameters” should be identified. Hyperparameters are a group of tuneable variables related to the architecture of the model and not learned from the dataset (unlike “parameters” which refer to the group of variables that are learned from the dataset during the training process). In our study, in order to determine the best hyperparameters, a grid search was performed for each model based on its performance, reflected by the accuracy. In addition, to validate the results and assure better generalisability, a tenfold cross-validation was performed. Finally, the best model was extracted to be tested on the testing set.

#### Training and testing

To predict to which extent maternal and newborn healthcare providers felt protected in the workplace, two sets of experiments were conducted. In each set, several ML models were trained and tested for a particular task.

##### Experiments set 1: classification models

*Experiment 1A—classification with all features.* Several classification models (Logistic Regression, SVM, RF, XGBoost, CatBoost, ANN) were trained and tested to predict a discrete class of the output describing the feeling of protection of the healthcare providers during the pandemic. After training and testing the models, features’ importance was extracted from the RF model, to determine the features that contribute the most to the prediction of the feeling of protection among maternal and newborn healthcare providers in the pandemic.

*Experiment 1B—classification with selected features.* Experiment 1A was repeated using only the top 10 selected features from the RF model for training. The RF was used for two reasons: (1) its tree-based strategy naturally ranks the features by how well they maximise the gain of information (or minimise the error) and contribute to the prediction, and (2) because it’s widely used in the literature for feature selection, especially for medical applications [44, 45]. This experiment was conducted to compare the performance of the models when trained using only the 10 most important features, to that of the models trained using all 71 features.

##### Experiments set 2: regression models

*Experiment 2A—regression.* Several regression models (Linear Regression, SVM, RF, XGBoost, CatBoost and ANN) were trained and tested to predict a decimal score from 1 to 5 reflecting the feeling of protection among healthcare providers during the pandemic. Features’ importance is once again extracted from the RF Regression model after training and compared to the results obtained in Experiment 1A.

*Experiment 2B—regression with selected features.* Experiment 2A was repeated using only the top 10 selected features from the RF model for training.

#### Performance metrics

Accuracy was used to evaluate and compare the performance of the classification models (Experiments 1A and 1B). The accuracy was chosen instead of the F1 score since the data became balanced after oversampling. On the other hand, root mean square error (RMSE) was used as a performance metric for the regression models (Experiments 2A and 2B). Equations (1) and (2) show the mathematical equations of accuracy and RMSE, respectively.

*Equation 1* *. Mathematical formula for accuracy*

$$Accuracy= \frac{Number \, of \, correct \, predictions}{Total \, number \, of \ predictions}.$$

*Equation 2* *. Mathematical formula for RMSE*

$$RMSE=\sqrt{\frac{1}{N} \sum_{i=1}^{N}{({y}_{i}-\widehat{{y}_{i}})}^{2},}$$

where $N$ is the number of elements in the sample, ${y}_{i}$ is the true value of the output of the *i*th element and $\widehat{{y}_{i}}$ is the predicted value of the output of the *i*th element.

## Results

### Sample description

The 941 respondents included in this analysis were from high-income countries (73%), middle-income (22%) and low-income countries (5%). The complete distribution of respondents across the 89 unique countries is available in Additional file 3. Table 1 displays the characteristics of respondents by country income group. Overall, half of respondents were midwives/nurse-midwives/nurses (50%), followed by obstetricians/gynaecologists (27%), and neonatologists/paediatricians (13%). About a third of respondents provided both inpatient and outpatient care services, and 22% provided at least two inpatient care services. More than half the respondents were team members (54%), followed by head of team (12%), head of department or ward (10%) and head of facility (6%). Overall, the majority of respondents were female (80%).

**Table 1:** Characteristics of the respondents (*n* = 941)

	HIC; *n* (%)	MIC; *n* (%)	LIC; *n* (%)	Total; *n* (%)
*Cadre*				
Midwife / nurse midwife / nurse	407 (59.4)	54 (26.2)	14 (28)	475 (50.4)
Obstetrician/gynaecologist	129 (18.8)	98 (47.6)	25 (50)	252 (26.8)
Neonatologist /paediatrician	111 (16.2)	15 (7.2)	1 (2)	127 (13.5)
General practitioner/medical doctor/intern	25 (3.6)	25 (12.1)	7 (14)	57 (6)
Other	13 (1.8)	14 (6.9)	3 (6)	30 (3.1)
*Type of care provided*				
Inpatient care only—1 service	127 (18.5)	28 (13.6)	5 (10)	160 (17)
Inpatient care only—2 or more services	185 (27.0)	22 (10.7)	3 (6)	210 (22.3)
Outpatient care only—1 or more services	26 (3.8)	14 (6.8)	3 (6)	43 (4.6)
Inpatient and outpatient care	193 (28.2)	92 (44.7)	25 (50)	310 (32.9)
Home visits and any inpatient or outpatient care	92 (13.4)	8 (3.9)	5 (10)	105 (11.2)
Home visits or community outreach	6 (0.9)	7 (3.4)	5 (10)	18 (1.9)
Community outreach and any inpatient or outpatient care	17 (2.5)	25 (12.1)	2 (4)	44 (4.7)
Home visits and community outreach and any other type of care	39 (5.7)	10 (4.9)	2 (4)	51 (5.4)
*Position*				
Head of facility (director, administrator)	23 (3.4)	29 (14.1)	3 (6)	55 (5.8)
Head of department or ward	45 (6.6)	36 (17.5)	14 (28)	95 (10.1)
Head of team	80 (11.7)	30 (14.6)	6 (12)	116 (12.3)
Team member	415 (60.6)	76 (36.9)	18 (36)	509 (54.1)
Locum or interim member	16 (2.3)	3 (1.5)	3 (6)	22 (2.3)
Other	106 (15.5)	32 (15.5)	6 (12)	144 (15.3)
*Gender*				
Female	595 (86.9)	132 (64.1)	23 (46)	750 (79.7)
Male	86 (12.6)	74 (35.9)	27 (54)	187 (19.9)
Prefer not say	4 (0.6)	0 (0)	0 (0)	4 (0.4)
Total	685 (100)	206 (100)	50 (100)	941 (100)

*HIC* high-income country, *MIC* middle-income country, *LIC* low-income country

Table 2 shows the distribution of respondents according to the characteristics of the facility where they primarily work. Most of the respondents worked in referral hospitals and district/regional hospitals (39% and 30%, respectively). About 70% of respondents worked in public facilities, and 15% worked in private facilities.

**Table 2:** Characteristics of the facilities where respondents’ mainly work (*n* = 941)

	HIC; *n* (%)	MIC; *n* (%)	LIC; *n* (%)	Total; *n* (%)
*Healthcare level of the institution where they work*				
Referral hospital	243 (35.5)	94 (45.6)	33 (66)	370 (39.3)
District/regional hospital	253 (36.9)	32 (15.5)	5 (10)	290 (30.8)
Health centre	51 (7.4)	26 (12.6)	4 (8)	81 (8.6)
Polyclinic/clinic/health post	83 (12.1)	30 (14.6)	4 (8)	117 (12.5)
Other (including self-employed or independent respondents)	55 (8)	24 (11.7)	4 (8)	83 (8.8)
*Sector type of the institution where they work*				
Public (national)	241 (35.2)	51 (24.8)	21 (42)	313 (33.3)
Public (university or teaching)	145 (21.2)	45 (21.8)	14 (28)	204 (21.7)
Public (district level or below)	104 (15.2)	19 (9.2)	4 (8)	127 (13.5)
Social security / health insurance	36 (5.2)	5 (2.4)	0 (0)	41 (4.4)
Private	90 (13.1)	51 (24.7)	5 (10)	146 (15.5)
NGO / faith based	23 (3.3)	26 (12.7)	5 (10)	54 (5.8)
Other	46 (6.7)	9 (4.4)	1 (2)	56 (6)
*Geographic area type*				
Large city (> 1 million inhabitants)	227 (33.1)	111 (53.9)	33 (66)	371 (39.4)
Small city (100,000 to 1 million inhabitants)	253 (36.9)	50 (24.3)	9 (18)	312 (33.2)
Town (< 100,000 inhabitants)	155 (22.6)	20 (9.7)	3 (6)	178 (18.9)
Village/rural area	43 (6.3)	19 (9.2)	2 (4)	64 (6.8)
Other	7 (1)	6 (2.9)	3 (6)	16 (1.7)
*Facility provides caesarean sections*				
No	71 (10.4)	34 (16.5)	5 (10)	110 (11.7)
Yes	607 (88.6)	171 (83)	44 (88)	822 (87.4)
Don't know	7 (1)	1 (0.5)	1 (2)	9 (1)
*Facility has intensive care unit for women w/ obstetric complications*				
No	194 (28.3)	73 (35.4)	18 (36)	285 (30.3)
Yes	488 (71.2)	131 (63.6)	31 (62)	650 (69.1)
Don't know	3 (0.4)	2 (1)	1 (2)	6 (0.6)
*Facility has neonatal intensive care unit*				
No	257 (37.5)	73 (35.4)	14 (28)	344 (36.6)
Yes	426 (62.2)	130 (63.1)	35 (70)	591 (62.8)
Don't know	2 (0.3)	3 (1.5)	1 (2)	6 (0.6)
*Facility receives maternity referrals from other facilities*				
No	208 (30.4)	40 (19.4)	2 (4)	250 (26.6)
Yes	471 (68.8)	164 (79.6)	48 (96)	683 (72.6)
Don't know	6 (0.9)	2 (1)	0 (0)	8 (0.9)
*Water and soap always available for staff*				
No	5 (0.7)	13 (6.3)	10 (20)	28 (3)
Yes	679 (99.1)	192 (93.2)	40 (80)	911 (96.8)
Don't know	1 (0.1)	1 (0.5)	0 (0)	2 (0.2)
*Water and soap always available for patients and visitors*				
No	24 (3.5)	31 (15)	18 (36)	73 (7.8)
Yes	653 (95.3)	171 (83)	32 (64)	856 (91)
Don't know	8 (1.2)	4 (1.9)	0 (0)	12 (1.3)
*Water and disinfectant always available to clean surfaces*				
No	17 (2.5)	26 (12.6)	19 (38)	62 (6.6)
Yes	662 (96.6)	177 (85.9)	29 (58)	868 (92.2)
Don't know	6 (0.9)	3 (1.5)	2 (4)	11 (1.2)
Total	685 (100)	206 (100)	50 (100)	941 (100)

*HIC* high-income country, *MIC* middle-income country, *LIC* low-income country, *NGO* non-governmental organisation

**Fig. 1:**
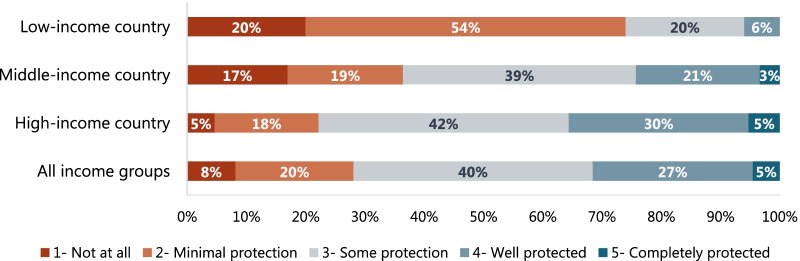
Perception of being protected in the workplace among maternal and newborn healthcare providers during the COVID-19 pandemic, by country income group

Figure 1 shows the distribution of respondents by perception of being protected in the workplace, on a 5-point Likert scale, by country income group. The majority of respondents in low-income countries (74%) reported feeling minimally or not at all protected, whereas this was reported by 36% and 22% of respondents from middle- and high-income countries, respectively. None of the respondents in low-income countries reported feeling completely protected, whereas this was reported by 3% and 5% of respondents in middle- and high-income countries.

### Hyperparameters

Table 3 summarises the set of hyperparameters for different models. Note that for the XGBoost and CatBoost models, the default hyperparameters were used. The ANN used is a multilayer perceptron composed of four layers: an input layer with one neuron for each feature (total of 71 neurons), two hidden layers with 120 neurons each and Relu activation function, and an output layer. Categorical cross-entropy loss with Adamax optimizer are set, and a batch size of 32 with 150 epochs are used for training.

**Table 3:** Set of hyperparameters used for support vector machine and random forest models in experiments 1A, 1B and 2

Model	Hyperparameters for Exp. 1A and 1B	Hyperparameters for Exp. 2A and 2B
Support Vector Machine	C = 1, kernel = ‘rbf’, gamma = ‘scale’	C = 1, kernel = ‘rbf’, gamma = '0.1’
Random Forest	Nb_estimators = 600, criterion = “gini”, max_depth = 15	Nb_estimators = 300, criterion = “mse”, max_depth = 25
XGBoost	Nb_estimators = 100, gamma = 0, max_depth = 6, learning_rate = 0.3, reg_lambda = 1	Nb_estimators = 100, gamma = 0, max_depth = 6, learning_rate = 0.3, reg_lambda = 1
CatBoost	Iterations = 1000, depth = 6, learning_rate = 0.08	Iterations = 1000, depth = 6, learning_rate = 0.04

### Experiment 1: classification

#### Experiment 1A: classification with all features

**Fig. 2:**
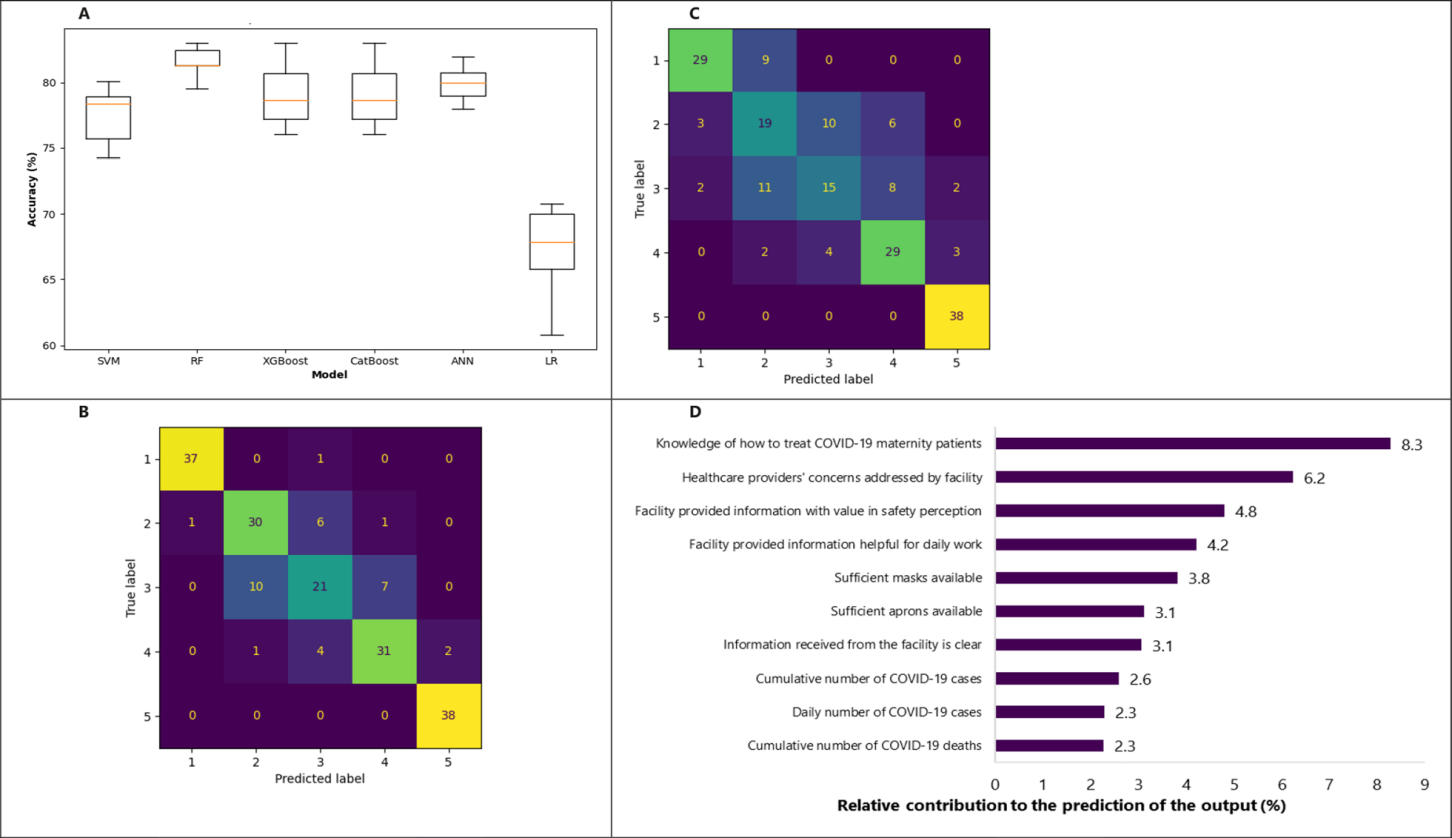
Visualisation of the results from Experiment 1A. **A **Boxplot of the tenfold cross-validated accuracies of different machine learning models (*SVM*  Support Vector Machine, *RF*  Random Forest, *ANN*  Artificial Neural Network, *LR*  Logistic Regression). **B **Confusion matrix of the random forest model on the testing set. **C **Confusion matrix of the logistic regression model on the testing set. **D **List of top 10 features by percentage relative contribution to the classification process, extracted from the random forest model

Figure 2A illustrates the accuracies of different ML models obtained by tenfold cross-validation, using a subset of the training set as a validation set. The best performing model was the RF (82%) followed by the ANN (80%), XGBoost (79%), CatBoost (79%) and SVM (78%). All models demonstrated better performance when compared to the conventional statistical technique, i.e. Logistic Regression, with an accuracy of 68%. This also applies to the performance on the testing set (the set of data that was obtained initially from the training–testing split and was never used during the training process), shown in Table 4.

**Table 4:** Accuracies of different models from Experiment 1A (classification with all features) on the testing set

Model	Accuracy (%) on testing set
Logistic Regression	68
Support Vector Machine	72
Random Forest	83
XGBoost	77
CatBoost	82
Artificial Neural Network	80

If we examine the confusion matrix of the RF model on the testing set (Fig. 2B), we notice that the number of missed classifications is low for classes 1 (“Not at all protected”) and 5 (“Completely protected”): 1 out of 38 (2.6%) and 0, respectively. This percentage progressively increased when we move centrally (to the middle classes) to achieve 45% of erroneous predictions for individuals belonging to class 3 (“Some Protection”). When compared to the Logistic Regression, the same pattern of distribution of misclassifications was present with overall higher percentages of error: 24% and 0% for classes 1 and 5, respectively, and the percentage increased centrally with up to 61% of missed classifications for class 3 (Fig. 2C). In addition, more predictions in the LR model are missing the class by more than 1 class (e.g. predicts 1 or 5 instead of 3), than with the RF: 12 versus 3, respectively. For instance, in class 3, we had 4 predictions that missed by 2 classes in the LR models, versus none in the RF model.

##### Feature extraction

The RF model was used to extract features that contributed the most to the classification. Figure 2D shows a list of features sorted based on their relative contribution to the classification of the output. The most salient feature was the knowledge of what to do in case of receiving a maternity patient confirmed with COVID-19. Other important features were: respondent reporting that their facility addressed concerns of healthcare providers, the perception that that the information provided by their health facilities has value in making respondents feel safe, is helpful in their daily work, and is clear, availability of sufficient PPE (masks, aprons), the cumulative and daily number of COVID-19 cases at the national level at the time of the survey, and the cumulative number of deaths due to COVID-19 at the national level. In other words, those features were found to be the primary predictors for classification.

#### Experiment 1B: classification with selected features

**Fig. 3:**
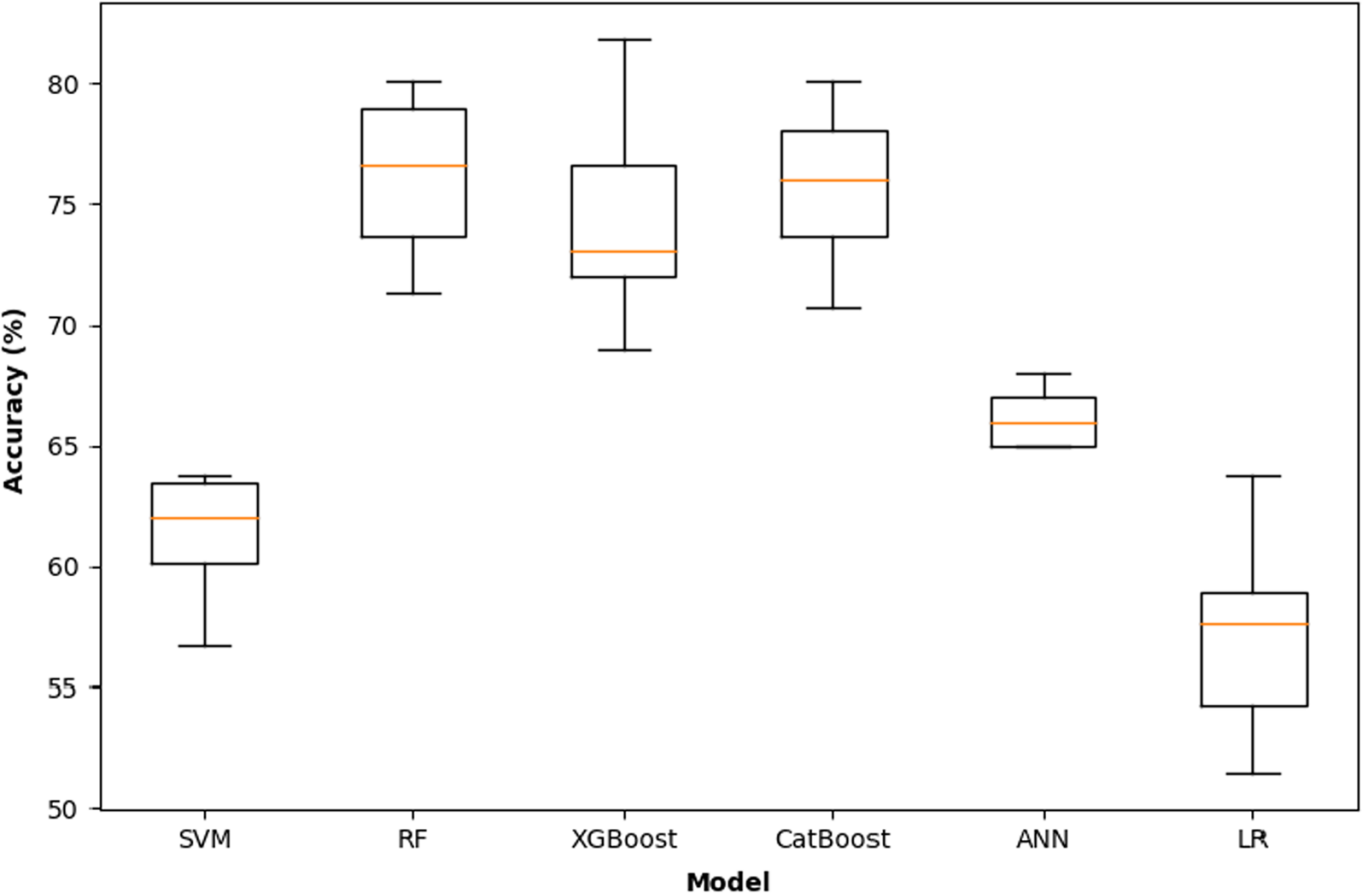
Boxplot of the tenfold cross-validated accuracies of different machine learning models, from Experiment 1B—classification with selected features (*SVM*  Support Vector Machine, *RF* Random Forest, *ANN*  Artificial Neural Network, *LR*  Logistic Regression)

Figure 3 and Table 5 show the tenfold cross-validated and the testing set accuracies of different machine learning models, respectively, after training the model using only the 10 most important features extracted from the RF model in experiment 1A (classification with all features).

**Table 5:** Accuracies of different models from Experiment 1B (classification with selected features) on the testing set

Model	Accuracy (%) on testing set
Logistic Regression	61
Support Vector Machine	62
Random Forest	77
XGBoost	81
CatBoost	76
Artificial Neural Network	65

Based on these results, the RF, XGBoost and CatBoost were the top performing models with tenfold cross-validated accuracies ranging from 74 to 77% and testing accuracies ranging from 76 to 81%; whereas, the ANN and SVM models scored lower with 65% and 62%, respectively. However, all the models performed better than LR that had a cross-validated accuracy of 57%.

When compared to the results of experiment 1A (classification with all features), the models were less performant when they were trained using only top 10 features rather than the entire dataset with 71 features. However, not all the models were affected the same way. In fact, the LR model was affected with a drop of 11% in accuracy (from 68% in experiment 1A to 57% in experiment 1B), the SVM with a drop of 16% and the ANN with a drop of 15%. On the other hand, the drop was generally much smaller in the RF, XGBoost and CatBoost models: 6%, 5% and 3%, respectively, which was expected since we the feature selection was based on the RF and since XGBoost and CatBoost also use tree-based strategy for classification.

### Experiment 2

#### Experiment 2A: regression with all features

**Fig. 4:**
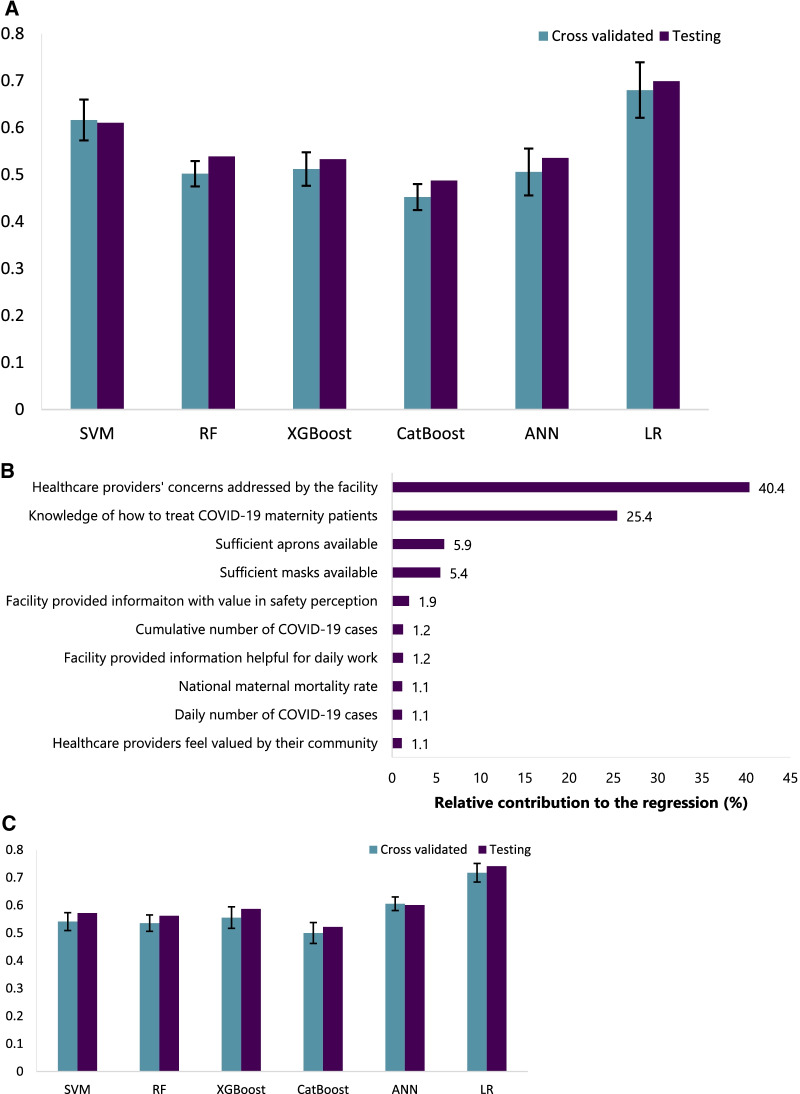
Visualisation of the findings from Experiments 2A and 2B. **A **Bar graph of the tenfold cross-validated RMSE and the testing set RMSE of different machine learning models, from Experiment 2A. **B **List of top 10 features by importance of contribution to the regression, extracted from the Random Forest Model in Experiment 2A. **C **Bar graph showing the tenfold cross-validated RMSE and the testing set RMSE of different machine learning models, from Experiment 2B. (*RMSE*  root mean square error, *SVM*  Support Vector Machine, *RF*  Random Forest, *ANN* Artificial Neural Network)

Figure 4.A shows the tenfold cross-validated RMSE (using each time a subset of the training set as a validation set) and the testing set RMSE (testing set obtained by train-test split and never used during training) of Linear Regression and machine learning models. The tenfold cross-validated RMSE of the Logistic Regression was 0.65. However, it was lower (reflecting a better performance) for all machine learning models, ranging between 0.46 for Catboost and 0.61 for SVM.

##### Feature extraction

Just like in Experiment 1A (classification with all features), top features were extracted from the RF Regression model, and are shown in Fig. 4B, sorted based on their contribution to the predictions. The two most important features for regression were facilities addressing the concerns of healthcare providers and knowledge of what to do in case of receiving a maternity patient confirmed with COVID-19. Other features included availability of sufficient PPE, facilities providing information that are considered helpful by respondents, the perception that healthcare providers are valued by their community, the national MMR, the level of healthcare of the institution, and the cumulative number of COVID-19 cases and deaths of the country at the time of the survey. The least contributing features include restrictions applied at the country-level, country income group, whether the facility received referrals or has an intensive care unit, and the respondents’ gender.

The classification and regression models yielded almost the same list of salient features, with slight changes in ranking.

#### Experiment 2B: regression with selected features

Figure 4C shows the tenfold cross-validated and the testing set RMSE of different machine learning models, after training the model using only the 10 most important features extracted from the RF model in experiment 2A (regression with all features). Even when trained on a subset of the features, machine learning models, with cross-validated RMSE ranging between 0.5 for CatBoost and 0.6 for ANN, outperformed Logistic Regression, with cross-validated RMSE of 0.72.

When compared to the results of Experiment 2A (regression with all features), all the models are less performant when trained on 10 features only, except the SVM which improves (RMSE of 0.53 vs 0.61 in Experiment 1A (classification with all features)).

## Discussion

This study was conducted by a multidisciplinary team representing computer engineering/science, clinical sciences, and public health. Therefore, our interpretations cover two distinct areas: lessons about the application of the ML method and implications for maternal health service provision. We discuss both in turn.

This study explored the potential for using ML models to predict the perception of being safe in the workplace among maternal and newborn healthcare providers during the COVID-19 pandemic. Our analysis shows that ML models perform better than conventional statistical methods in terms of accuracy and margins of error. This was the case for all the models across different experiments, with the RF, XGBoost and CatBoost being the most robust models. By analysing the confusion matrices of the Logistic Regression model and the RF model from experiment 1A (classification with all features), we notice that (1) ML models (and particularly RF in this case) have overall a better performance when compared to the conventional techniques and are less likely to make large class prediction deviation, and (2) the likelihood of misclassification errors in the prediction process increases as we move to the middle class (i.e. class 3). This has an important significance on the interpretation of the Likert scale output, as the feeling of being protected is a subjective perception that results, just like any other human perception, from the complex interaction between environmental, genetic, biologic, and psychosocial factors, and this complexity is difficult to capture accurately in surveys. Despite that, ML models, due to their architecture and algorithms, are capable of more accurately capturing these interactions and this explains why the number of erroneous predictions is lowest for individuals belonging to class 0 (Not at all protected) and 5 (Completely Protected), and highest for individuals belonging to class 3 (Some Protection), because the former are certain about their feeling while the latter have already a certain level of uncertainty.

Experiment 1B (classification with selected features) also shows that some ML models (RF, XGBoost and CatBoost) are capable of making accurate predictions when trained on a small number of features without losing much accuracy, which is not the case for conventional statistical models. This is particularly important because it allows the use of such a tool to screen for the perception of feeling protected among healthcare providers without needing to collect a large number of features (fewer questions).

Experiments 2A (regression with all features) and 2B (regression with selected features), on the other hand, attempt to solve the same problem using regression. These experiments are implemented for several reasons. First, by considering the output as a continuous variable, we are capable of representing the perception of being protected as a spectrum which is more realistic than the discrete categories. Second, this allows to quantify the exact amount of error at the individual level to avoid under or overestimation of the model’s performance. For instance, if the classification model predicts 2 instead of 3, we cannot detect how far the model was from making the correct prediction, whereas in the regression model, we are able to quantify the error. Third, re-iterating the problem using a different ML model, contributes to confirming the validity of the models when similar results are obtained from the various models; which was the case in this study. The results of the experiments show that even when the problem is solved using regression, ML models are more robust at making the predictions than conventional techniques, with a mean error of 0.5 class.

By applying the RF algorithm, we are able to extract and rank features by the extent to which they contribute to the prediction of healthcare providers’ feeling of protection in the workplace. The findings from both experiments were cross-validated by comparing the features’ rankings between both experiments. The top ten features in both experiments 1A (classification with selected features) and 2A (regression with selected features) were classified in three main themes: (1) information accessibility, clarity and quality; (2) availability of support and means of protection; and (3) COVID-19 epidemiology at the national level. The three themes are discussed below in detail.

### 1—Information accessibility, clarity and quality

Features belonging to this theme include healthcare providers’ knowledge on what to do in case of having a COVID-19 maternity case (ranked 1 and 2, respectively, in both experiments), and healthcare providers’ perception of the information that they received from the facility regarding COVID-19 and maternity care (in terms of value in feeling safe, helpfulness in daily work, and clarity). This suggests that access to information and knowledge, particularly clear information and feasible recommendations, plays a key role in the morale of maternal and newborn healthcare providers. Our results also highlight that the quality of the information received relative to each healthcare providers’ needs and perceptions, has an important contribution to healthcare providers’ attitudes and wellbeing. Previous studies, at global and national levels, show that healthcare providers struggled with the lack of knowledge, guidance and prevailing uncertainty during the early days of the pandemic [15, 17, 30]. Particularly in the case of maternity care, global guidelines and recommendations took some time to be established, and evidence regarding the risk of COVID-19 for women and newborn continues to emerge to this day [46]. This lack of clarity can be stressful for those providing care to women and newborns in these uncertain circumstances [47], and be translated as perceptions of unsafety when providing care. On the other hand, some facilities established clear guidelines on referring women with confirmed COVID-19 to other facilities or to COVID-19 treatment centres. This could have contributed to a perception of low exposure to COVID-19 risks among healthcare providers working in the referring facilities and consequently a perception of protection in the workplace. Future studies exploring whether differences in perception of protection exist between healthcare providers who work in facilities that refer COVID-19 obstetric cases and those who treat them on site.

### 2—Availability of support and means of protection

Two main features were grouped to represent the support received from the health facility where healthcare providers work: whether the facility addressed their concerns (ranked 2 and 1, respectively, in both experiments), and the availability of sufficient PPE (masks and aprons). Healthcare providers are a core building block of the healthcare system, and providing quality care can only be achieved when human resources are empowered and supported. The healthcare system must be responsive and adaptive to the needs of its workforce and therefore able to address their concerns and worries, regularly and in times of crises [48]. Globally, PPE shortage was a significant issue in the early days of the pandemic for all cadres of healthcare providers. Essential healthcare providers such as maternal and newborn care workers who were not caring directly for COVID-19 patients, may have experienced this shortage more acutely, as they might not have been prioritised to receive PPE and had to continue providing clinical care. Research showed that this was a source of concern for maternal and newborn healthcare providers as many of them worried about their own safety and becoming infected with COVID-19 in the workplace as a result of the lack of PPE [9, 12, 14, 17]. Additionally, the mere availability of PPE is not sufficient, and maternal and newborn healthcare providers must have access to appropriate support and training on PPE use. This includes training on adequate donning and doffing, as well as learning to provide empathic care while wearing them [14, 47]. In our survey, these questions were specific to support received from the health facility where respondents worked. Nonetheless, it is worth mentioning that the support that health facilities can provide is conditional upon the support and resources that facilities receive from higher structures in the healthcare system, nationally and globally. For example, facilities cannot ensure PPE availability to care providers if there is a national and global shortage. Additionally, facilities cannot communicate guidelines and information to frontline care providers unless those have been officially issued by health authorities. Therefore, the interpretation of these features as a responsibility of health facilities should be made with caution, as we consider the responsiveness of health facilities to be a mere reflection of the responsiveness of the healthcare system.

### 3—COVID-19 epidemiology at the national level

Features grouped under this theme represent the level of spread of the COVID-19 outbreak at the country level including the cumulative number of COVID-19 cases and deaths due to COVID-19 and the daily number of cases reported on the day of data collection. Our results show that the extent of the transmission of the virus contributes to the prediction of healthcare providers’ perception of protection in the workplace. Healthcare providers, much like the rest of the community, are sensitive to these kinds of changes at the national level, and it is reflected in their attitudes in the workplace. The higher the number of COVID-19 cases and deaths in the community, the higher the likelihood of having to provide care to women confirmed or suspected with COVID-19. This influences the level of risk perceived by healthcare providers and their perceptions of being protected in the workplace. These values are publicly available data at the national level, making the prediction of the output at the individual level easier to achieve.

### Least contributing factors

Further analysis reveals that restriction measures applied at the national-level are among the least contributing factors to the prediction of the outcome. In a previous analysis using qualitative data from the same survey conducted at a time point further into the pandemic, we identified that maternal and newborn healthcare providers’ perception of being safe was linked to the extent of the COVID-19 restrictions applied at the country-level [49]. However, the results from the current quantitative analysis contradict our qualitative findings. This shows that ML analysis, although can be valuable in informing a rapid response, can be supplemented by qualitative data in order to represent a clearer, more in-depth assessment of the wellbeing of healthcare providers in emergency situations. The country-income group also had a minimal contribution in predicting healthcare providers’ safety feeling. This highlights the need to consider healthcare providers’ wellbeing in various context, particularly considering the gap in research conducted in low- and middle-income countries on this issue. Some facility-level characteristics, such as the reception of referrals or the presence of an intensive care unit were also among the least contributing factors to the outcome. Although higher level facilities have been given the responsibility to handle COVID-19 cases in many countries, healthcare providers in lower level facilities have had similar experiences of safety perception as those working in higher level facilities. The gender of the healthcare providers was also a minimally contributing factor to the perception of safety feeling. This finding may warrant further exploration in future studies designed to unpack gendered differences in the impact of the pandemic on maternal and newborn healthcare providers, the majority of whom are women.

### Strengths and limitations

This is one of the first studies that uses ML to develop an algorithm that predicts maternal and newborn healthcare providers’ feeling of protection in the workplace during the early phases of the COVID-19 pandemic, using data collected through an online survey. This work is one of the few applications of ML models to subjective survey data, and despite the large number of limitations and assumptions associated with analysing “perceptions and opinions” quantitatively, the results are promising and the method has a relatively high level of accuracy (81%).

Nonetheless, with the application of ML in public health research, the results must not be taken at face value, and must be interpreted with caution [50]. To ensure the relevance of our findings beyond numbers, and to confirm the validity of the applied methods we adopted two approaches: (1) a cross-comparison of the features identified in two experiments, which shows that most features exist in the top 10 across the two experiments (convergent validity); and (2) and a thorough qualitative interpretation of the top-ranked features contributing to the prediction of the output in light of pre-existing literature and knowledge, which supported/confirmed the conceptual validity of the tool. This process highlights the importance of the multidisciplinary collaboration between computer engineering/science and public health, which leveraged the value of the work and validated the findings from different perspectives.

One possible limitation of our work is that additional features that could have contributed to the prediction of the output were absent from the analysis. This includes information that was not initially collected in the survey such as personal features (e.g. age, years of experience, experience with previous outbreaks and disruptive events) and individual risk-factors for COVID-19. Other information was collected in the survey but in an open-ended manner, and therefore were not included in this analysis, such as being re-assigned to COVID-19 treatment wards, being diagnosed/suspected to have COVID-19, colleagues diagnosed with COVID-19 or the number of deaths due to COVID-19 among healthcare providers at the country level, etc. Future applications of this tool should consider expanding the list of features, including an additional feature on the availability of COVID-19 vaccines to healthcare providers.

The study’s sampling technique and online data collection meant that the data are not representative of the healthcare provider population, and we acknowledge the potential of a selection bias given that there was no sampling frame for the global study participants. Additionally, many respondents to the original sample were excluded from the final analysis because they had incomplete fields or missing information, which could have affected the sampling bias. Information bias could also exist in the data, particularly related to the quality of reporting national estimates of the number of COVID-19 cases and deaths.

The scope of our work and survey and research area is limited to maternal and newborn healthcare providers. There is potential to evaluate such advanced methods in research related to other cadres of healthcare providers, including those who are at the frontline of providing care to COVID-19 patients.

This study provides factors that predict the perception of safety among a global sample of healthcare providers who work in different settings. It was not possible to assess context-specific factors that could predict the outcome differentially based on the country setting or income-group because of the small size of the sub-samples. Future developments of ML models at the country-level can unpack context-specific factors that can be addressed at the local level, particularly for low- and middle-income countries.

Finally, it is important to mention that we do not underestimate the utility and importance of conventional techniques, but rather embrace both techniques and take advantage of their strengths based on the problem to be solved. For instance, for some problems with small datasets, conventional techniques offer a fast and cost-effective solution, whereas for complicated problems with large datasets and nonlinear interaction between different variables, machine learning algorithms might offer a better alternative.

## Conclusion

The COVID-19 pandemic has challenged health systems globally, not only by having to respond to an overwhelming number of COVID-19 cases, but also by having to adjust quickly to severe restriction measures and their impact on the health workforces (quarantine, isolation, sickness or death, inability to reach the workplace, etc.). Our study shows that both pandemic-related and health system-related factors contributed significantly to maternal and newborn healthcare providers’ perceptions of feeling safe during the pandemic. According to the WHO quality of care framework, “competent and motivated human resources” are essential for ensuring quality care to women and newborns [51]. It is critical to prioritise the wellbeing of maternal and newborn healthcare providers, by ensuring they have adequate access to up-to-date, clear, and practical information, and essential means of protection during the COVID-19 pandemic [47].

The tool developed in this study can have two applications: on an individual level, it can inform the development of a future screening tool for perceptions of being safe among maternal and newborn healthcare providers; and it could be used as a simulation model to assess the impact of personal, facility-based, health systems related and policy-level measures on the perception of being safe among maternal and newborn healthcare providers.

The latter application can be used in healthcare settings (either in health facilities or within professional organisations) to guide policy and planning during shocks to the healthcare systems, including the ongoing COVID-19 pandemic. This tool could have the ability to better leverage real-time insights and translate them to preventive interventions efficiently and rapidly, with a specific focus on the wellbeing of healthcare providers [50]. By responding in real-time to the needs of healthcare providers, the health system could prevent potential negative consequences on the quality of care offered to women and newborns.

## Supplementary Information

**Additional file 1.**  Complete list of features used in the models.

**Additional file 2.** Description of different ML algorithms.

**Additional file 3.**  Distribution of respondents across countries.

## Data Availability

Due to ethical constraints, the data underlying this analysis cannot be made publicly available. The dataset cannot be completely de-identified without removing key variables such as country, cadre, facility level, facility sector, area type. This de-identification would limit the value of the dataset, making any replication of the analysis impossible. Data requests can be sent to the study PI Prof Lenka Benova at lbenova@itg.be and the ethics committee at the Institute of Tropical Medicine at irb@itg.be.

## Abbreviations

AI: Artificial intelligence
ANN: Artificial Neural Network
HIC: High-income country
LIC: Low-income country
ML: Machine learning
MMR: Maternal mortality ratio
MERS: Middle east respiratory syndrome
MIC: Middle-income country
PPE: Personal protective equipment
RF: Random Forest
SARS: Severe acute respiratory syndrome
SVM: Support Vector Machine
SMOTE: Synthetic minority oversample technique
UK: United Kingdom
WHO: World Health Organization

## Declarations

### Patient and public involvement

Patients and/or the public were not involved in the design, or conduct, or reporting, or dissemination plans of this research.
