# Supplementary material for: Can machine learning models predict maternal and newborn healthcare providers’ perception of safety during the COVID-19 pandemic? A cross-sectional study of a global online survey
Source: Hum Resour Health. 2022 Aug 19;20:63. doi: 10.1186/s12960-022-00758-5 (PMC9389509; doi:10.1186/s12960-022-00758-5)
Supplement: Supplementary file 1 — Additional file 1. Complete list of features used in the models. [file 12960_2022_758_MOESM1_ESM.docx]

**Part 1. Features extracted from the MatCO online survey**

| *Feature* | | *Response options* |
| --- | --- | --- |
| In which **country** are you based (providing healthcare) at the moment? | | [drop down menu of countries] |
| What is your **position**?  (choose one) | | - Head of facility (director, administrator) - Head of department or ward - Head of team - Team member - Locum or interim member - Other: specify |
| What is your **gender**?  (choose one) | | - Female - Male - Other/Prefer not to say |
| What **type of maternal and/or neonatal health care** do you currently provide as an individual? | | - Inpatient care – only 1 service - Inpatient care – 2 or more services - Outpatient care– 1 or more services - Inpatient and outpatient care services - Home visits and any inpatient or outpatient service - Home visits or community outreach only - Community outreach and any inpatient or outpatient service - Home visits and community outreach and any other type of service |
| In which **level** of health care institution do you primarily work?  (if none of the response options fit well, please use the “Other” option and write what your facility type is called in your country) | | - Referral hospital - District/regional hospital - Health center - Polyclinic - Clinic - Health post/unit - Dispensary - Other: specify |
| What organisation **type** is your institution? | | - Public (national) - Public (university or teaching) - Public (district level or below) - Social security - Health insurance or HMO - Private university - Private for profit - Non-governmental - Faith-based or mission - Other: specify |
| In what type of **geographic area** is your facility located? | | - Large city (>1 mil inhabitants) - Small city (100,000 to 1 mil inhabitants) - Town (<100,000 inhabitants) - Village or rural area - Refugee or displaced persons camp - Other |
| Does your facility provide **caesarean sections**? | | Yes  No |
| Does your facility have an **Intensive Care Unit (ICU)** which can admit **women** with obstetric complications? (ICU is defined as a clinical area where ventilatory support can be provided) | | Yes  No  Don’t know |
| Does your facility have a **neonatal intensive care unit (NICU)**?  (Neonatal intensive care is defined as a unit that provides invasive ventilatory support to small and sick newborns, not just CPAP) | | Yes  No  Don’t know |
| Does your facility **receive maternity referrals** from other facilities, meaning that patients are sent to your facility from other health facilities? | | Yes  No  Don’t know |
| Is running **water and soap** always available for hand hygiene on your ward **for the use of staff**? | | Yes  No  Don’t know |
| Is running **water and soap** always available on your ward for the use of **patients, visitors, companions**? | | Yes  No  Don’t know |
| Is there always **sufficient water and disinfectant for cleaning surfaces**? | | Yes  No  Don’t know |
| Has your institution or ward provided you with **any information** on how to prepare for COVID-19? | | Yes  No |
| On a scale from **1 (poor) to 5 (excellent)**, how would you rate the following dimensions of this information: | | 0 – No information provided  1 – poor  2 – somewhat useful  3 – average  4 – good  5 – excellent |
| Clarity | |  |
| Helpfulness for your daily work | |  |
| Value in helping you feel safe | |  |
| Has your institution or ward provided you with any training on COVID-19, for example simulations or drills? | | Yes  No |
| Have you received new or updated guidelines specifically for the provision of care to pregnant, labouring or postpartum women and their newborns because of COVID-19? | | Yes  No |
| Have you personally searched for source of guidance and sources of information to prepare for COVID-19 in your work? | | Yes  No |
| Have you received information related to COVID-19 and your work informally through other colleagues (in your own facility or outside)? | | Yes  No |
| Have you been a part of any self-organisation on the part of healthcare workers in response to the COVID-19 outbreak? (exchange of information, virtual discussion groups (Whatsapp, Facebook, etc.) | | Yes  No |
| Has your facility published or distributed any materials (brochure, flier, posters, etc.) covering COVID-19 targeted toward pregnant, labouring, or postnatal women? | | Yes  No  Don’t know |
| In your facility, do you feel that patients’ questions about COVID-19 are being addressed adequately by staff? | | Yes  No  Don’t know |
| Has your facility set up a well sign-posted general entrance and screening area for COVID-19 suspected cases?  (regardless whether for maternity patients or not) | | -Yes  -Some measures taken but not done well  -No measures taken  -Don’t know |
| Has your facility reserved **isolation rooms** for COVID-19 suspected cases? | | Yes  No  Don’t know |
| Has **routine cleaning of the maternity ward** changed in response to COVID-19? | | Yes, increased  Yes, decreased  Unchanged  Don’t know |
| Is your facility currently screening for COVID-19 symptoms among maternity patients with COVID-19? | Yes  No  Don’t know  Other: specify | |
| Is it possible to order a test for COVID-19 at the moment for maternity patients at your facility? | Yes  No  Don’t know  Other: specify | |
| Are the testing criteria for COVID-19 clear to you? (the conditions/symptoms for which a test can be ordered) | Yes  No | |
| Have you had any maternity patients with COVID-19 in your facility so far? | Yes, suspected  Yes, confirmed  No  Don’t know  Other: specify | |
| Is there a designated COVID-19 lead person / liaison in the maternity ward or the facility? | -Yes, in maternity  -Yes, in facility as a whole  -No, neither maternity nor facility  -Don’t know | |
| On a scale from 1 (not at all) to 5 (I am very clear), do you personally feel you know what you should do if a woman with COVID-19 symptoms arrives in your facility today? | 1 – Not at all clear  2 – Some points are clear to me, but I am not confident in what to do  3 – Somewhat clear but major issues remain  4 – I am mostly clear but some questions / areas of concern remain  5 – I am very clear | |
| On a scale from 1 (not at all) to 5 (completely), do you feel that you are sufficiently protected from infection with COVID-19 in your workplace? | 1 – not at all  2 –minimal protection  3 – some protection  4 – well protected  5 – completely protected | |
| Is a sufficient quantity of personal protective equipment (PPE) available to you? |  | |
| Gloves | Yes - No | |
| Masks | Yes - No | |
| Aprons | Yes - No | |
| Has your work been affected by the COVID-19 outbreak? | - Yes - No | |
| On a scale of 1 (not at all) to 5 (completely), do you feel that your concerns about the response to COVID-19 have been addressed by your facility or ward? | 1 – not at all  2 – minimally  3 – somewhat  4 – well  5 – completely | |
| How would you rate your own levels of stress at this time? | -Same as usual  -Somewhat higher than usual  -Substantially higher than usual | |
| Do you consider your personal role as a health worker in this COVID-19 outbreak is valued by the community you are serving? | Not at all  Very little  Somewhat  Highly  Unsure/don’t know | |

**Part 2. Features added from public sources**

| **Source** | **Feature** | **Definition/Options** |
| --- | --- | --- |
| WHO COVID-19 dashboard | Cases | Daily number of COVID-19 cases at the national level |
|  | Cumulative cases | Cumulative number COVID-19 cases at the national level on the day of data collection |
|  | Deaths | Daily number of deaths due to COVID-19 at the national level |
|  | Cumulative deaths | Cumulative number of deaths due to COVID-19 at the national level on the day of data collection |
| Oxford COVID-19 Government Response Tracker | Domestic lockdown | 0 - No  1 - Yes |
|  | Domestic local lockdown | Binary variable to distinguish localized domestic lockdowns from other cases. 1 denotes that domestic lockdowns were implemented at the local level and 0 means that domestic lockdowns were not implemented at the local-level (either at the national level or not implemented). |
|  | Travel restrictions | 0 - No  1 - Yes |
|  | Travel restrictions partial | Binary flag to differentiate partial travel restrictions from other cases. 1 denotes that travel restrictions were partial and 0 denotes that travel restrictions were not partial (either strict or not implemented). |
|  | Travel restrictions domestic | Binary variable equal to1 if travel restrictions within the country were implemented and 0 otherwise |
|  | Travel restrictions domestic partial | Binary flag to differentiate partial domestic travel restrictions from other cases. 1 denotes that travel restrictions were partial and 0 denotes that travel restrictions were not partial (either strict or not implemented). |
|  | Curfew | 0 - No  1 - Yes |
|  | Curfew partial | Binary flag to differentiate partial curfews from other cases. 1 denotes that the curfew was partial and 0 denotes that the curfew was not partial (either strict or not implemented). |
|  | Mass gatherings banned | 0 - No  1 - Yes |
|  | Mass gatherings banned partial | Binary flag to distinguish localized bans on mass gatherings from other cases. 1 denotes that bans on mass gatherings were partial and 0 denotes that bans on mass gatherings were not partial (either strict or not implemented). |
|  | Sports events and large event banned | 0 - No  1 - Yes |
|  | Sports events and large event banned partial | Binary flag to distinguish partial bans and cancellations of sporting and large events. 1 denotes that bans on sporting and large events were localized, strict or with no spectators, 0 that bans on sporting and large events are not localized or partial (either national or no measures implemented). |
|  | Restaurants closed | 0 - No  1 - Yes |
|  | Restaurants closed local | Binary flag to distinguish localized and/or partial restaurant and bar closures from other cases. The variable is coded 1 in the three following situations: localized closures, limitations on the number of customers in bars and restaurants, and closures of either bars or restaurants. 0 indicates national closures or no closures at all. |
|  | Public testing policy | 0 - No  1 - Yes |
|  | Public testing policy narrow | Binary flag to distinguish narrow testing policies from large testing policies. 1 denotes that testing policies were targeted to some individuals, 0 that testing policies were not targeted (either large or not implemented). |
|  | Obligation to wear masks | 0 - No  1 - Yes |
|  | Obligation to wear masks partial | Binary variable equal to1 if the obligation to wear masks is regional. When the measure was regional, was coded 1. On the contrary, when the measure was national, was coded 0. |
|  | Surveillance | Binary variable equal to 1 if mobile app or bracelet surveillance was implemented and 0 otherwise. |
|  | Surveillance partial | Binary variable equal to1 if the enhanced surveillance is optional or reserved for a category of person (e.g. certain professions or foreigners) and 0 otherwise. |
|  | Rigidity public health | Average of the thirteen coded public health measures. Public health measures are valued 0.5 if they are localized or partial and 1 if they are national or strict. 0 indicates no measures. The index is computed if there are at least 10 out of the 13 measures coded. |
|  | Population size | Population size estimate in 2019 |
|  | Continent | Continent to which the country belongs |
| World Bank 2019 estimates | GDP per capita | Per capita values for gross domestic product (GDP) expressed in current international dollars converted by purchasing power parity (PPP) conversion factor.   - GDP is the sum of gross value added by all resident producers in the country plus any product taxes and minus any subsidies not included in the value of the products. - Conversion factor is a spatial price deflator and currency converter that controls for price level differences between countries. - Total population is a mid-year population based on the de facto definition of population, which counts all residents regardless of legal status or citizenship. |
|  | Country income group | High income country  Middle income country  Low income country |
| WHO 2017 estimates | Maternal mortality ratio | Maternal mortality per 100,000 live births |
