# Supplementary material for: Can machine learning models predict maternal and newborn healthcare providers’ perception of safety during the COVID-19 pandemic? A cross-sectional study of a global online survey
Source: Hum Resour Health. 2022 Aug 19;20:63. doi: 10.1186/s12960-022-00758-5 (PMC9389509; doi:10.1186/s12960-022-00758-5)
Supplement: Supplementary file 2 — Additional file 2. Description of different ML algorithms. [file 12960_2022_758_MOESM2_ESM.docx]

**Additional file 2 - Description of different ML algorithms**

The SVM is a powerful classification tool widely used in the health field. It works by searching for a decision boundary (also known as a hyperplane) which maximizes the margins between the classes of the output. Its robustness is reflected by many of its properties. First, SVM does not impose restrictions on the distribution of the data to be used. Second, the decision boundary is a function of some selected points from each class (known as support vectors) which makes it an appropriate algorithm for datasets with high number of parameters and low number of data points. And finally, SVM is capable of handling datasets with nonlinear relations (nonlinearly separable datasets) by applying a kernel transformation to the dataset to map the elements into higher dimensions that render the dataset linearly separable.^[[1]](#endnote-1)^

RF is an ensemble of decision trees that classify data points by splitting the dataset into subsets in a way to maximize the information gain at each split. It combines several decision trees, using the “bagging method”, to obtain a more accurate and generalizable prediction.^[[2]](#endnote-2)^ It is one of the most applied ML algorithms because of its capability to perform both classification and regression tasks. Furthermore, RF is capable of extracting the relative importance of different features in the prediction thus helping researchers acquire some insight about the relations of parameters in their dataset, and eventually drop the features that are not relevant for the output prediction.

XGboost and Catboost are also tree-based algorithms. But unlike RF, they combine several decision trees using the “boosting method”. In this method, the trees learn in a sequential fashion, where early trees perform simple and easy predictions, and subsequent trees are executed to enhance the performance and boost the number of correct predictions.

Finally, the ANN is an algorithm inspired by the structure of the brain. It consists of multiple neurons organized in several layers. These neurons are interconnected with weights that are adjusted during the learning process, and that determine the final output of the model. They are organized in layers: an input layer where the independent variables (features) are fed to the model, one or more hidden layers where data is processed and weights are determined, and an output layer that gives the dependent variable (output). There are several types of ANN, but in this paper, we used the Multilayer Perceptron Neural Network which is a feedforward neural network where different layers of the model are fully connected (each neuron of a certain layer is connected to all neurons of the following layer). ANN is widely used because it does not impose any restrictions on the distribution of the data, can handle large datasets and can learn and model complex and nonlinear relations due to its architecture.^[[3]](#endnote-3)^

1. Saxe GN, Ma S, Ren J, Aliferis C. Machine learning methods to predict child posttraumatic stress: a proof of concept study. BMC psychiatry. 2017;17(1):1-13; Boser BE, Guyon IM, Vapnik VN. A training algorithm for optimal margin classifiers. Paper presented at: Proceedings of the fifth annual workshop on Computational learning theory 1992. [↑](#endnote-ref-1)
2. Breima L. Random Forests. Machine Learning. 2010. [↑](#endnote-ref-2)
3. Renganathan V. Overview of artificial neural network models in the biomedical domain. Bratislavske lekarske listy. 2019;120(7):536-540. [↑](#endnote-ref-3)
