# Supplementary material for: Can machine learning models predict maternal and newborn healthcare providers’ perception of safety during the COVID-19 pandemic? A cross-sectional study of a global online survey
Source: Hum Resour Health. 2022 Aug 19;20:63. doi: 10.1186/s12960-022-00758-5 (PMC9389509; doi:10.1186/s12960-022-00758-5)
Supplement: Supplementary file 3 — Additional file 3. Distribution of respondents across countries. [file 12960_2022_758_MOESM3_ESM.docx]

**Country distribution – High-income countries**

| **Country** | **n(%)** |
| --- | --- |
| Australia | 13 (1.9) |
| Austria | 6 (0.88) |
| Barbados | 1 (0.15) |
| Belgium | 43 (6.28) |
| Canada | 29 (4.23) |
| Chile | 14 (2.04) |
| Cyprus | 2 (0.29) |
| Denmark | 3 (0.44) |
| Estonia | 1 (0.15) |
| France | 21 (3.07) |
| Germany | 26 (3.8) |
| Greece | 1 (0.15) |
| Iceland | 4 (0.58) |
| Ireland | 4 (0.58) |
| Israel | 1 (0.15) |
| Italy | 150 (21.9) |
| Japan | 60 (8.76) |
| Lithuania | 1 (0.15) |
| Luxembourg | 1 (0.15) |
| Malta | 1 (0.15) |
| Netherlands | 5 (0.73) |
| New Zealand | 5 (0.73) |
| Norway | 1 (0.15) |
| Portugal | 3 (0.44) |
| Singapore | 2 (0.29) |
| Slovakia | 2 (0.29) |
| Spain | 13 (1.9) |
| Sweden | 16 (2.34) |
| Switzerland | 20 (2.92) |
| Trinidad and Tobago | 2 (0.29) |
| United Kingdom | 38 (5.55) |
| United States | 45 (6.57) |
| Uruguay | 151 (22.04) |
| **Total** | **685 (100)** |

**Country distribution – Middle-income countries**

| **Country** | **n (%)** |
| --- | --- |
| Argentina | 16 (7.77) |
| Bangladesh | 11 (5.34) |
| Benin | 1 (0.49) |
| Bhutan | 1 (0.49) |
| Bolivia | 2 (0.97) |
| Brazil | 4 (1.94) |
| Bulgaria | 1 (0.49) |
| Cambodia | 1 (0.49) |
| Cameroon | 4 (1.94) |
| Colombia | 1 (0.49) |
| Ecuador | 1 (0.49) |
| El Salvador | 1 (0.49) |
| Georgia | 1 (0.49) |
| Ghana | 5 (2.43) |
| Guatemala | 2 (0.97) |
| Guyana | 1 (0.49) |
| Honduras | 2 (0.97) |
| India | 44 (21.36) |
| Indonesia | 2 (0.97) |
| Iraq | 4 (1.94) |
| Jordan | 1 (0.49) |
| Kenya | 13 (6.31) |
| Laos | 1 (0.49) |
| Lebanon | 2 (0.97) |
| Mexico | 2 (0.97) |
| Moldova | 2 (0.97) |
| Morocco | 6 (2.91) |
| Namibia | 1 (0.49) |
| Nepal | 1 (0.49) |
| Nigeria | 17 (8.25) |
| Pakistan | 1 (0.49) |
| Panama | 9 (4.37) |
| Papua New Guinea | 1 (0.49) |
| Paraguay | 1 (0.49) |
| Peru | 1 (0.49) |
| Philippines | 9 (4.37) |
| Senegal | 1 (0.49) |
| South Africa | 3 (1.46) |
| Suriname | 1 (0.49) |
| Tanzania | 18 (8.74) |
| Thailand | 1 (0.49) |
| Uzbekistan | 1 (0.49) |
| Vietnam | 1 (0.49) |
| Zambia | 5 (2.43) |
| Zimbabwe | 2 (0.97) |
| **Total** | **206 (100)** |

**Country distribution – Low-income countries**

| **Country** | **n (%)** |
| --- | --- |
| Afghanistan | 2 (4) |
| Ethiopia | 4 (8) |
| Guinea | 2 (4) |
| Malawi | 1 (2) |
| Mozambique | 11 (22) |
| Niger | 1 (2) |
| Rwanda | 5 (10) |
| Sierra Leone | 1 (2) |
| The Gambia | 1 (2) |
| Togo | 1 (2) |
| Uganda | 21 (42) |
| **Total** | **50 (100)** |
